# Short- and Long-Term Mortality in Severe Sepsis/Septic Shock in a Setting with Low Antibiotic Resistance: A Prospective Observational Study in a Swedish University Hospital

**DOI:** 10.3389/fpubh.2013.00051

**Published:** 2013-11-15

**Authors:** Anna Linnér, Jonas Sundén-Cullberg, Linda Johansson, Hans Hjelmqvist, Anna Norrby-Teglund, Carl Johan Treutiger

**Affiliations:** ^1^Division of Infectious Diseases, Center for Infectious Medicine, Karolinska Institutet, Karolinska University Hospital, Stockholm, Sweden; ^2^Department of Anesthesiology, Karolinska Institutet, Karolinska University Hospital, Stockholm, Sweden

**Keywords:** severe sepsis, septic shock, mortality, gender, antibiotics

## Abstract

**Background**: There is little epidemiologic data on sepsis, particularly in areas of low antibiotic resistance. Here we report a prospective observational study of severe sepsis and septic shock in patients admitted to the Intensive Care Unit (ICU) at Karolinska University Hospital, Sweden. We aimed to evaluate short- and long-term mortality, and risk factors for sepsis-related death. A second aim was to investigate patient care in relation to gender.

**Methods**: One hundred and one patients with severe sepsis and septic shock, admitted to the ICU between 2005 and 2009, were prospectively enrolled in the study. Defined primary endpoints were day 28, hospital, and 1-year mortality. Risk factors for sepsis-related death was evaluated with a multivariate analysis in a pooled analysis with two previous sepsis cohorts. In the subset of patient admitted to the ICU through the emergency department (ED), time to clinician evaluation and time to antibiotics were assessed in relation to gender.

**Results**: In the septic cohort, the day 28, hospital, and 1-year mortality rates were 19, 29, and 34%, respectively. Ninety-three percent of the patients received adequate antibiotics from the beginning. Multi-resistant bacteria were only found in three cases. Among the 43 patients admitted to the ICU through the ED, the median time to antibiotics was 86 min (interquartile range 52–165), and overall 77% received appropriate antibiotics within 2 h. Female patients received antibiotics significantly later compared to male patients (*p* = 0.047).

**Conclusion**: The results demonstrate relatively low mortality rates among ICU patients with severe sepsis/septic shock, as compared to reports from outside Scandinavia. Early adequate antibiotic treatment and the low incidence of resistant isolates may partly explain these findings. Importantly, a gender difference in time to antibiotic therapy was seen.

## Introduction

Despite better understanding of the pathophysiology and improved management of sepsis, severe sepsis, and septic shock, the incidence continues to increase (1–5). The mortality rates still remain unacceptably high, ranging from 31 to 61% in multicentre trials of septic shock (6–10).

Efforts have been made during the past decade to develop protocols for management, in analogy with the previous evolution of standardized care of other acute medical conditions like myocardial infarction and multitrauma (11–14). An outcome benefit is associated with early diagnosis and structured resuscitation of patients with severe sepsis and septic shock, i.e., Early-Goal-Directed-Therapy (EGDT) (12, 15, 16). Elapsed time from triage and qualification for EGDT to administration of appropriate antimicrobials are primary determinants of mortality in patients with severe sepsis and septic shock (17). Kumar et al. (18) reported that correct and rapid administration of adequate antibiotics significantly reduces mortality rates, with each hour of delay in antimicrobial administration over the ensuing 6 h being associated with an average decreased survival of 7.6%. However, it was recently emphasized that in patients who received antibiotics after shock recognition, there is no increase in mortality associated with a delay in antibiotics administration (19).

Levy et al. (20) recently reported a significant difference between the USA and Europe in unadjusted mortality in severe sepsis/septic shock patients. Importantly, this difference disappeared when adjusting for severity of illness, emphasizing the complexity in interpreting results from different sites. Several factors including among others regional health care approaches as well as access to care, age, and comorbid disease burden will all influence outcome. In Scandinavian countries lower mortality rates are commonly reported, for instance in the Finnsepsis study the Intensive Care Unit (ICU), hospital, and 1-year mortality rates in severe sepsis were 15.5, 28.3, and 40.9%, respectively (21). The Finnsepsis study group also reported low mortality rates in community-acquired septic shock with ICU and hospital mortalities of 22 and 35%, respectively (22). In Norway, hospital mortality in severe sepsis and septic shock were 27 and 29%, respectively (23). Likewise, we have in our previous studies on biomarkers in sepsis observed a relatively low day 28 mortality rate of 15.6 and 26% in severe sepsis and septic chock (24, 25). The Scandinavian countries share a beneficial antibiotic susceptibility setting that allows for studies of clinical outcome in severe infections not complicated by antibiotic resistance.

The aim of this paper was to investigate day 28, hospital, and 1-year mortality in a prospective study of hospital and community-acquired severe sepsis and septic shock patients treated in an ICU in a Swedish University Hospital, and to evaluate risk factors for sepsis-related death. A second aim was to investigate patient care in the emergency department (ED), in particular time to antibiotics and time to clinician evaluation in relation to gender.

## Materials and Methods

### Study setting and patient cohorts

In this prospective observational cohort study, a total of 101 patients were included in the septic cohort during the study period from October 2005 to June 2009. Inclusion criteria were a diagnosis of severe sepsis or septic shock within the last 24 h in patients admitted to the mixed medical and surgical ICU (12 beds) of Karolinska University Hospital Huddinge, a tertiary care facility. Because of hospital sub-specialization in Stockholm County, the Karolinska University hospital in Huddinge does not admit multi-trauma patients and patients with elective lower GI, thoracic, or brain surgery are operated elsewhere. In the septic cohort, 43 patients were admitted to the ICU through the ED, allowing for evaluation of immediate patient care. Identification and enrollment of patients was performed by a research nurse during day-time, Monday to Friday recruitment, which explains the inclusion of patients over an extended period of time.

Severe sepsis and septic shock, including the clinical variables were defined according to the criteria proposed by the American College of Chest Physicians/Society of Critical Care Medicine (26). The diagnosis of sepsis required a clinical assessment of infection together with systemic inflammatory reaction. Systemic inflammatory reaction was defined by at least two of the following criteria: fever or hypothermia (temperature >38.0 or <36.0°C, respectively), tachycardia (heart rate >90 beats/min), tachypnea (respiratory rate >20 breaths/min, or PaCO2 <32 torr (4.3 kPa) or the requirement of mechanical ventilation), and white blood cell count >12 × 10^9^/L (or >10% immature white blood cells). Severe sepsis was defined as sepsis in addition to signs of acute reduction of organ perfusion (not related to primary septic focus or underlying chronic disease) as manifested by at least one of the following: (a) acute deterioration of mental status; (b) arterial hypoxemia [PaO2 <75 torr (10 kPa) without evidence of primary lung disease]; (c) oliguria (urine production <0.5 mL/kg/h for >2 h); (d) acute deterioration of liver function (*S*-bilirubin >43 μmol/L, or *S*-alanine transaminase more than twice elevated above reference value; (e) metabolic acidosis (plasma lactate elevated above normal levels or base excess ≥5 mEq/L); or (f) recent coagulation abnormality (prothrombin time or activated partial thromboplastin time ≥1.2 times the upper limit plus D-dimer ≥0.5 mg/L or platelets ≤75 × 10^9^/L or a 50% reduction in 24 h). Septic shock was defined as severe sepsis in addition to hypotension requiring vasopressor support, or mean arterial pressure <70 mmHg for ≥30 min despite adequate fluid resuscitation.

In addition to the prospective cohort above, we also included sepsis patients enrolled in two previous prospective studies as a confirmatory cohort for comparison of mortality rates. These studies had similar or identical inclusion criteria and were conducted at the same study site between 1998 and 2001 (*n* = 54; 22 severe sepsis and 32 septic shock) and 2003 and 2005 (*n* = 50; 9 severe sepsis and 41 septic shock) (24, 25).

The study was conducted in accordance with the declaration of Helsinki and was approved by the local ethics committee of Karolinska University Hospital. Written informed consent was obtained from the patients or their close relatives, and are archived by the authors.

### Data collection and outcomes

Blood samples were collected from all patients and controls at inclusion (0 h). Tubes were immediately centrifuged at 3000 rpm for 10 min and aliquots of plasma were stored at −70°C until analysis. Standard laboratory analyses were performed at the Clinical chemistry laboratory, Huddinge, according to the manufacturer’s instructions.

Clinical data, including variables specified in Table 1, were registered according to a predesigned Case Record Form daily for all patients until day 7. Severity of disease was measured by Acute Physiology and Chronic Health Evaluation (APACHE) II (27) at admittance and also by daily Sepsis-related Organ Failure Assessment (SOFA) scores until day 7 (28). These scores and final diagnoses were determined retrospectively, on the basis of complete patient charts and laboratory tests. The results of blood and microbiological cultures were recorded.

**Table 1:** **Baseline patient characteristics and laboratory findings in total cohort, and survivors vs. non-survivors**.

Variable	Septic cohort*, *n* = 101	Survivors, *n* = 82	Non-survivors, *n* = 19	*p*-Value
**GENDER, AGE**				
Male, *n* (%)	55 (55)	43 (52)	12 (63)	0.452
Age, median (range)	64 (23–89)	64 (23–89)	64 (45–86)	0.449
**SEVERITY OF DISEASE, MEAN ± SD**				
SOFA 0 h	10 ± 3.4	9.6 ± 3.2	11.6 ± 4.1	0.054
SOFA 24 h	8.9 ± 3.7	8.3 ± 3.6	11.7 ± 2.8	0.0004
SOFA 96 h	5.2 ± 3.9	5.2 ± 3.5	5.4 ± 5.5	0.767
APACHE II	22.7 ± 8.0	21.0 ± 7.4	26.7 ± 9.8	0.016
**ACQUISITION OF INFECTION, *n* (%)**				
Community	62 (61)	51 (62)	11 (58)	0.796
Hospital	39 (39)	31 (38)	8 (42)	0.796
**UNDERLYING CONDITIONS, *n* (%)**				
Previously healthy, *n* (%)	11 (11)	9 (11)	2 (11)	1.0
Diabetes	16 (16)	13 (16)	3 (16)	1.0
Smoking	15 (15)	10 (12)	5 (42)	0.151
Alcohol abuse	11 (11)	7 (9)	4 (21)	0.212
History of cancer	31 (31)	22 (27)	9 (47)	0.100
Immunosuppression^a^	27 (27)	19 (23)	8 (42)	0.146
Hypertension	24 (24)	23 (28)	1 (5)	0.038
Heart disease^b^	21 (21)	17 (21)	4 (21)	1.0
Pulmonary^c^	3 (3)	3 (4)	0 (0)	0.573
Liver disease^d^	8 (4)	4 (5)	4 (21)	0.039
Neurologic disease^e^	5 (5)	3 (4)	2 (11)	0.236
Renal failure	4 (4)	3 (4)	1 (5)	0.572
Other diseases^f^	15 (15)	12 (15)	3 (16)	1.0
Blood cultures (*n* = 92)		76	16	
Confirmed positive (%)	50 (54)	40 (53)	10 (63)	0.585
**CLINICAL MANIFESTATIONS, *n* (%)**				
Pneumonia	25 (28)	17 (21)	8 (80)	0.075
Urinary tract infection	16 (16)	14 (17)	2 (10)	0.729
Intra-abdominal infection	42 (42)	36 (44)	6 (60)	0.440
Skin/soft tissue infection	9 (9)	7 (9)	2 (10)	0.676
Neutropenia	4 (4)	3 (4)	1 (5)	0.572
Bacterial meningitis	3 (3)	3 (4)	0 (0)	1.0
Undefined origin	2 (2)	2 (2)	0 (0)	1.0
**LABORATORY FINDINGS, MEDIAN (IQR)**				
C-reactive protein (mg/L)	226 (140–308)	227 (143–316)	210 (89–276)	0.224
WBC (10^9^/L)	14.3 (5.6–23.7)	14.3 (5.9–21.9)	13.6 (0.9–27.2)	0.664
Hemoglobin (g/L)	105 (92.5–117.5)	104.5 (96.5–118)	106 (99–113)	0.651
Platelet count (10^9^)	165 (82–265)	171.5 (83–266)	126 (66–213)	0.262
P-glucose (mmol/L)	7.0 (6.1–8.9)	7.1 (6.4–9.0)	6.4 (4.5–7.3)	0.031
aPTT (s)	46 (40.5–56)	45.5 (40–53.3)	52 (44–62)	0.082
Creatinine (μmol/L)	155 (89–246.5)	153 (86–250)	163 (108–225)	0.357
Bilirubin (μmol/L)	18 (9–30.5)	16 (8–30)	23 (15–54)	0.058
INR	1.4 (1.2–1.8)	1.4 (1.2–1.7)	1.6 (1.2–2.7)	0.043
D-dimer (mg/L)	1.9 (0.8–3.9)	1.5 (0.8–3.7)	3.3 (1.4–7.2)	0.063
Lactate (mmol/L)	3.2 (2.2–5.2)	3 (2.1–4.0)	7.6 (2.8–11.1)	0.004

*Septic cohort consisted of 86 patients with septic shock and 15 patients with severe sepsis.

*^a^ According to APACHE II criteria*.

*^b^ A history of heart disease contains of coronary artery disease and congestive heart failure*.

*^c^ Chronic obstructive pulmonary disease or emphysema or asthma*.

*^d^ Liver disease contains of hepatitis with or without cirrhosis and primary biliary cirrhosis*.

*^e^ Neurologic diseases: previous stroke and multiple sclerosis*.

*^f^ Inflammatory bowel disease, rheumatoid arthritis, psychiatric disease, ventricular ulcer, or hypothyreos*.

The primary outcomes studied were day 28 mortality, in-hospital mortality (death during hospital stay), and 1-year mortality. The outcomes were further compared with the above described independent cohorts. The multivariate regression analysis for risk factors of death in severe sepsis and septic shock was performed and the survival rate was further analyzed in groups stratified by age. We also retrospectively registered adequate antibiotic treatment (determined based on comparison between administered antibiotics and blood culture findings and/or diagnosis) and the presence of multi-resistant bacteria defined as Extended Spectrum Beta Lactamase (ESBL) or other multi-resistant Gram-negatives, Vancomycin Resistant *Enterococci* (VRE), or Meticillin Resistant *Staphylococcus aureus* (MRSA). We retrospectively studied the timing of antibiotic administration and clinician evaluation in those patients that were admitted to the ICU through the ED (*n* = 43) and performed a comparison based on gender. At Karolinska University Hospital the exact time (hours:minutes) of the above specified factors as well as time of admission is recorded in the electronic patient chart.

### Statistical analysis

Descriptive data are presented as mean (SD) for continuous data, and medians with interquartile ranges (IQRs) for numerous data that did not follow Gaussian distribution. To test for normality, we used recommended D’Agostino and Pearson omnibus normality test. Comparisons between groups were made by the non-parametric Mann–Whitney *U* test, or for categorical values, Fisher’s exact test. A two-tailed *p*-value <0.05 was considered statistically significant. The survival analysis was made by using Kaplan–Meier survival curve. The analysis of risk factors for death in the septic cohort was performed using a multivariate cox regression analysis performed in two steps. In the first step, univariate analyses were performed for demographic and clinical factors listed in Table 3. All factors with a univariate *p*-value of <0.1 were entered into a stepwise Cox regression model where the model selection was based on the Akaike Information Criteria (AIC) approach. The GraphPad Prism 5 (GraphPad Software, La Jolla) was used for all statistical analyses except the multivariate analysis where the R version 2.14.1 was used.

## Results

### Patients: Clinical and microbiological characteristics

After final classification, 86 patients with septic shock and 15 patients with severe sepsis were included in the study, i.e., a total number of 101 patients referred to as the septic cohort.

Clinical characteristics and laboratory findings are presented in Table 1. In each group of patients, the majority (>89%) had underlying medical conditions, such as a history of cancer, cardiac disease, and diabetes. In addition to cardiovascular shock, 49% of septic shock patients had kidney failure, 84% had respiratory failure, and 15% had coagulation abnormalities. All septic shock patients were put on vasopressor support, 93% were on insulin therapy at study inclusion, and four patients were treated with intravenous immunoglobulin adjunctive therapy. Thirty-eight patients were immunosuppressed according to APACHE II criteria. High-dose cortisone (i.e., exceeding a dose of 1000 mg of hydrocortisone daily) was given to only one patient with septic shock, whereas 73% of septic shock and 60% of severe sepsis patients received low dose cortisone (50–200 mg of hydrocortisone three to four times daily). The median ICU and hospital length of stays were 5 and 26 days, respectively, in the septic cohort.

The most prevalent underlying causes of severe sepsis and septic shock were abdominal infections, pneumonia, and urosepsis (Table 1), which together represented 82% of the septic cohort. Five patients had infections of undefined origin.

In all patients with severe sepsis and septic shock, blood cultures were taken in 92 out of the 101 patients and 49% of these were culture-positive. The highest frequency of blood cultures were obtained in patients with urinary tract infections (88% positive cultures), skin/soft tissue infections (77% positive cultures), and intra-abdominal infections (36% positive cultures). There was an overrepresentation of Gram-negative bacteria (59%) as compared to Gram-positive bacteria (41%), consistent with abdominal origin being the most prevalent source of infection. *Escherichia coli* was the pathogen most commonly isolated (Table 2), predominantly from patients with urinary tract or intra-abdominal infections. Etiology was established in another 38 cases through alternative microbiological cultures and serology. Fourteen patients in the septic cohort lacked conclusive microbiological diagnosis.

**Table 2:** **Microbiological findings of blood cultures**.

Organism	No. isolates (*n* = 49)
*Escherichia coli*	18
*Staphylococcus aureus* (MSSA^a^)	5
*Klebsiella pneumonia*	3
*Pseudomonas*	3
*Streptococcus pneumonia*	3
*Serratia marcescens*	2
*Enterococcus faecalis*	7
*Proteus mirabilis*	1
*Candida*	1
*Corynebacterium*	1
*Clostridium septicum*	1
*Peptostreptococci*	1
*Neisseria meningitides*	1
*Staphylococcus epidermidis*	1
*G*+ *cocci*^b^	1

*^a^ MSSA, meticillin-susceptible *S. aureus**.

*^b^ Not possible to identify further*.

In relation to microbiological findings or diagnosis, 93% of patients with severe sepsis or septic shock received adequate antibiotic treatment. Seven patients received suboptimal antibiotic treatment in relation to subsequent culture findings, but none of these had multi-resistant bacteria (i.e., ESBL, other multi-resistant Gram-negatives, VRE, or MRSA). Of the 101 patients in our septic cohort, only 2 had positive blood cultures of ESBL producing bacteria (*E. coli*), 1 had VRE, and 2 had *Serratia marcescens* resistant to Cefuroxim. The most common initial empiric antibiotic treatment was carbapenems (60%), followed by cephalosporins (20%), and piperacillin-tazobactam (13%). Twenty-three percent received concurrent treatment with aminoglycosides. Adequate antimicrobial therapy refers to empirical treatment on admittance, at which time most patients did not have a microbiological diagnosis. Many patients had postsurgical or hospital acquired infections, for which carbapenems are recommended in Swedish guidelines.

### Short- and long-term mortality, and risk factors for death

**Figure 1:**
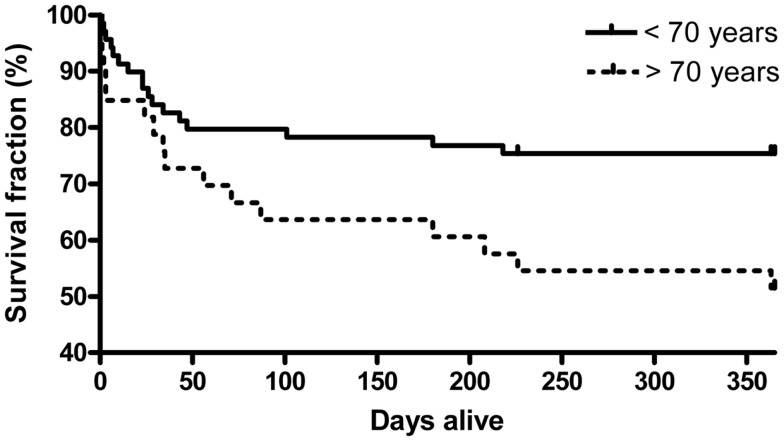
**Kaplan–Meier estimated survival in the septic cohort by age (<70, or 70 years and older)**. (*p*-Value = 0.021, log-rank test, χ^2^ = 5.36).

The overall day 28 mortality rate was 19% in the septic cohort and the hospital mortality was 29%. There were five additional deaths after discharge in the septic cohort, yielding 1-year mortality rate of 34%. Patients were stratified by age with a cut off at 70 years. As expected, the elderly patients (*n* = 33 vs. 68 in the younger group), had higher day 28, hospital, and 1-year mortality compared with the younger group (21, 33, and 48 vs. 18, 26, and 26%) (Figure 1).

The day 28 non-survivors all died of septic shock with multiple organ failure. The most common cause of death between days 28 and 365 was also a sustained septic shock with multiple organ failure followed by cardiac heart failure, pneumonia, cardiac arrest, acute myocardial infarct, and liver failure.

Two previous studies with independent cohorts of 54 and 50 patients with severe sepsis and septic shock, enrolled at the same site as the current study, reported low day 28 mortality rates (24, 25). Here, we retrospectively calculated the hospital and 1-year mortality of these previous cohorts. The hospital mortality rate was 33% and the 1-year mortality rate was 41%, consistent with our current study. Mortality rates in pooled data with all three cohorts were 22% at day 28 (*n* = 45/205) and 38% at day 365 (*n* = 77/205). The hospital mortality rate was 31% (*n* = 62/205).

A multivariate regression analysis for risk factors of death in severe sepsis and septic shock was performed using all patients in the three sepsis cohorts (*n* = 205, septic shock *n* = 159, severe sepsis *n* = 46). All independent factors with a *p*-value of <0.1 in the univariate analysis listed in Table 3 were included in the multivariate analyses in which age, cardiac heart failure, immunosuppression, and SOFA score were shown to be independent risk factors for death (Table 4).

**Table 3:** Risk factors for negative outcome in severe sepsis and septic chock*, univariate analysis with cox proportional hazards regression.

	Hazard ratio	Lower limit	Upper limit	*p*-Value
Gender	1.02	0.65	1.60	0.931
Age	1.03	1.01	1.04	0.003
Pneumonia	1.31	0.82	2.09	0.258
Abdominal infection	0.72	0.44	1.17	0.181
Urinary tract infection	0.70	0.32	1.53	0.377
Smoking	1.66	1.01	2.73	0.044
Alcohol	1.57	0.87	2.85	0.138
Diabetes	0.97	0.53	1.80	0.930
Coronary artery disease	0.67	0.31	1.46	0.314
Cardiac heart failure	1.97	1.04	3.74	0.037
Liver disease	2.02	1.07	3.83	0.031
Chronic obstructive lung disease	0.79	0.29	2.17	0.653
Immunosuppression^a^	1.93	1.22	3.04	0.005
Lactate level	2.01	1.25	3.22	0.004
SOFA score (day 1)	1.17	1.10	1.25	<0.001

*Patients from the current study and the two pooled comparative cohorts of severe sepsis and septic chock.

*^a^ According to APACHE II criteria*.

**Table 4:** Risk factors for negative outcome in severe sepsis and septic shock*, multivariate stepwise cox regression analysis.

	Hazard ratio	Lower limit	Upper limit	*p*-Value
Age	1.02	1.00	1.04	0.019
Smoking	1.62	0.97	2.71	0.063
Cardiac heart failure	2.21	1.12	4.36	0.022
Liver disease	1.93	1.00	3.74	0.050
Immunosuppression^a^	2.41	1.50	3.87	<0.001
Lactate	1.67	1.00	2.77	0.049
SOFA score (day 1)	1.12	1.05	1.20	0.001

*Patients from the current study and the two pooled comparative cohorts of severe sepsis and septic shock.

*^a^ According to APACHE II criteria*.

### Patients with community-acquired severe sepsis/septic shock admitted to the ICU through the ED

**Figure 2:**
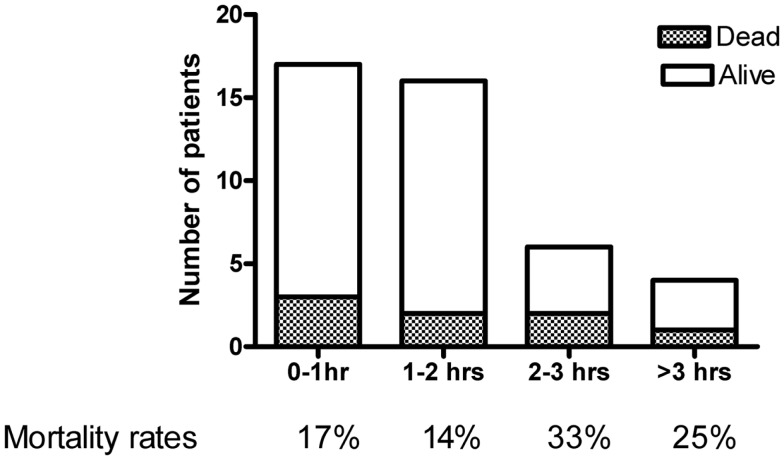
**Time to adequate antibiotics in hours, and relation to mortality rate**. The figure includes severe sepsis and septic shock patients (*n* = 43) admitted to the ICU directly from the emergency department.

The septic cohort included 43 patients with community-acquired severe sepsis and septic shock arriving to the ICU directly from the ED (Table 5). This cohort was used to specifically assess time to see a clinician and to antibiotic administration. In the total cohort, the immediacy of fluid treatment was also registered. Nine patients received pre-hospital (ambulance) fluid treatment. The remaining 34 received fluid after a mean 46 min (range 0–157 min), whereof 21% within 15 min, 47% within 30 min, and 71% of the patients within 60 min. Pre-ICU fluids consisted only of crystalloids, colloids were only given in the ICU. Sixty-three percent of the patients were assessed by a physician within 30 min, 98% within 60 min. The median time to first dose of antibiotic treatment was 86 min (IQR 52–165). Seventy-three percent of the patients received antibiotic treatment within 2 h. Mortality rates within each hour of antibiotic delay is shown in Figure 2.

**Table 5:** **Sepsis patients admitted to ICU through emergency department (ED): baseline patient characteristics in total cohort and in female vs. male patients**.

Variable	Septic ED cohort*, *n* = 43	Female, *n* = 17 (40%)	Male, *n* = 26 (60%)	*p*-Value
**AGE**				
Age, median (range)	64 (23–89)	61 (26–76)	71 (23–89)	0.013
**MORTALITY, *n* (%)**				
28-day mortality	8 (19)	4 (24)	4 (15)	0.692
1-year mortality	13 (30)	5 (29)	8 (31)	1.0
**SEVERITY OF DISEASE, MEAN ± SD**				
SOFA 0 h	10 ± 3.3	9.9 ± 3.8	10 ± 3.1	0.970
APACHE II	24.1 ± 8.5	22.3 ± 8.9	25 ± 8.2	0.249
**UNDERLYING CONDITIONS, *n* (%)**				
Previously healthy, *n* (%)	11 (26)	3 (18)	8 (31)	0.480
Diabetes	7 (16)	2 (12)	5 (19)	0.685
Smoking	1 (2)	0 (0)	1 (4)	1.0
Alcohol abuse	7 (16)	2 (12)	1 (4)	0.685
History of cancer	10 (23)	5 (29)	5 (19)	0.481
Immunosuppression^a^	10 (23)	5 (29)	5 (19)	0.481
Hypertension	14 (33)	5 (29)	9 (35)	1.0
Coronary artery disease	6 (14)	4 (24)	2 (8)	0.193
Chronic heart failure	3 (7)	1 (6)	2 (8)	1.0
**CLINICAL MANIFESTATION, *n* (%)**				
Pneumonia	17 (40)	7 (41)	10 (38)	1.0
Urinary tract infection	11 (26)	6 (35)	5 (19)	0.296
Intra-abdominal infection	4 (9)	0 (0)	4 (15)	0.140
Skin/soft tissue infection	6 (14)	2 (12)	4 (15)	1.0
Neutropenia	1 (2)	1 (6)	0 (0)	0.395
Bacterial meningitis	1 (2)	1 (6)	0 (0)	0.395
Undefined origin	3 (7)	0 (0)	3 (12)	0.266
Time to see a physician, median (IQR) (min)	20 (5–35)	32 (8–39)	12 (4–35)	0.219
Time to adequate antibiotics from triage, median (IQR) (min)	86 (52–165)	144 (70–185)	67 (36–135)	0.047
**CYTOKINES, MEDIAN (IQR)**				
IL-6 (pg/mL)	560 (106–3710)	1902 (120–6925)	477 (89–1373)	0.2097
IL-8 (pg/mL)	79 (31–1708)	200 (60–2362)	43 (23–890)	0.0729
IL-10 (pg/mL)	23 (9–67)	32 (9–248)	18 (9–53)	0.2912

*Septic cohort of 43 patients with 34 patients with septic shock and 9 patients with severe sepsis coming to the ICU through the ED.

*^a^ According to APACHE II criteria*.

**Figure 3:**
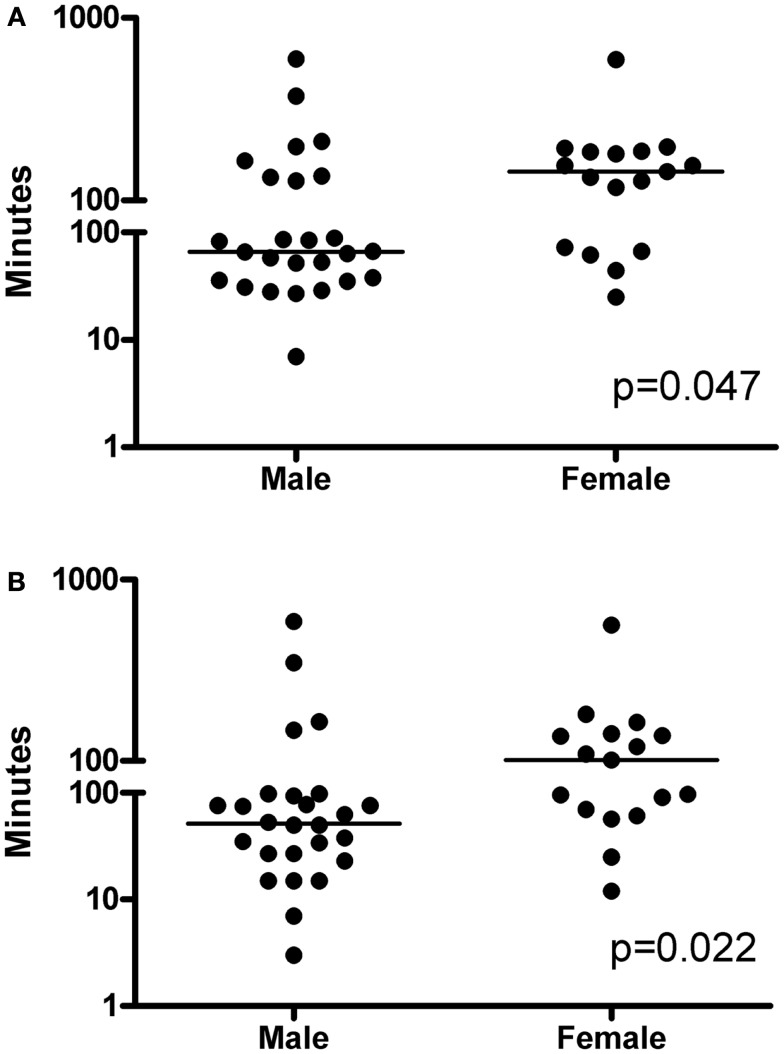
**Gender differences in (A) time from triage to antibiotics (*p* = 0.047) and (B) time from seeing the physician to adequate antibiotics (*p* = 0.022)**. Statistically significant differences between the groups were determined by the non-parametric Mann–Whitney *U* test. A two-tailed *p*-value <0.05 was considered statistically significant and the *p*-values are indicated. Horizontal lines denote median values.

The analyses were also made according to gender, and as shown in Table 5, the female patients were younger than the male patients (*p* = 0.013) but were similar with respect to severity of disease, diagnosis, and comorbidities. In addition, no significant difference in baseline cytokine levels were found, although female showed slightly higher levels of all three cytokines. Female patients waited for physician evaluation for a median time of 32 min (IQR 8–39 min) as compared to 12 min (IQR 4–35 min) after admission to the ED for male patients (*p* = 0.22). In addition, female patients had a significantly delayed time to antibiotic treatment as compared to men, a median of 144 min (IQR 70–185) compared to 67 min (IQR 36–135 min; *p* = 0.0469) (Figure 3A). Measuring time from physician assessment to first dose of antibiotics, we found similar gender differences. Women received the first dose at 101 min (IQR 66–140 min) as compared to 52 min (IQR 25–96 min) for males (*p* = 0.0216) (Figure 3B).

## Discussion

This prospective observational study of severe sepsis and septic shock in a mixed medical and surgical ICU in a Swedish University Hospital is based on a septic cohort, which is comparable with cohorts of previously reported international studies in respect to severity of sepsis, defined by severity scores, age, and underlying conditions (10, 18, 21). We report mortality rates of 19% (day 28), 29% (hospital), and 34% (1 year). Both short- and long-term mortality was due to a septic shock with multiple organ failure which in the late mortality cases were followed by cardiac heart failure, pneumonia, cardiac arrest, acute myocardial infarct, and liver failure. Risk factors for death were age, cardiac heart failure, immunosuppression, and SOFA score. The noted mortality rates are lower than that reported in the study by Kumar et al. (18), in which the hospital mortality rate amounted to 56% in septic shock patients, as well as in the study by Ranieri et al. (29) with a reported day 28 mortality rate of 25.3%. Similarly, in an European multicentre study of septic shock patients (10), in which ages and SOFA scores matched those of our study, the 28-day mortality rate was 33% and the hospital mortality rate was 43%. It is important to highlight that our septic cohort includes 15% severe sepsis patients whereas the others only had septic shock. However, inclusion of only septic shock patients from our cohort resulted in even lower mortality rates; day 28 mortality of 17% and hospital mortality of 29%. Our mortality rates are in line with those reported from Scandinavia such as in the Finnsepsis study (21).

As highlighted in the recent report by Levy et al. (20) many factors influence mortality rates in different settings, not the least different health care systems and approaches to critical care. In the Stockholm area, clinical subspecialties are largely administratively centralized to different hospitals, such as upper gastrointestinal surgery for which Karolinska University Hospital Huddinge is the main center; explaining the dominance of abdominal infections in our study, with *E. coli* being the most prevalent pathogen. There are few data related to the effects of different sources of infection on outcome. It has previously been suggested that abdominal infections may be more severe (30, 31) than respiratory infections. A recent study showed no differences in age, sex, severity score, or mortality rates between the two groups, but the development of septic shock, early coagulation, and acute renal failure was more common in patients with abdominal infections (32). The fact that abdominal infections dominate should, if anything, influence our mortality rates negatively.

Many studies have shown that the prognosis of severe sepsis and septic shock can be improved by using internationally recommended guidelines (33). The efficacy and speed of early management and adequate treatment in the initial hours after onset of illness are likely to influence outcome, in analogy with the treatment of acute myocardial infarction or acute trauma. Early Goal-Directed Therapy in severe sepsis and septic shock patients has been shown to improve 1-year mortality rate compared to standard treatment (34). Our 1-year mortality rate matches this outcome although there was no explicit adherence to an EGDT protocol. Importantly, the severity of disease in our septic shock patients was higher (SOFA score 10.4 ± 3.4 vs. 7 ± 4) suggesting that our patients were more severely ill compared to the patients in the study of Puskarich et al. (34). National guidelines in Sweden, at the time of the study recommended the use of low dose corticosteroids for septic shock patients in the need of inotropic support, but is currently restricted to those with persisting hypotension despite inotropic therapy. Seventy-three percent of our septic shock patients received hydrocortisone, indicating underprescription by guidelines at the time, but overprescription by current standards. Overprescription likely had no effect on mortality (10).

Time to receive adequate antibiotics is a critical survival factor (18), in particular prior to shock recognition (19). In this study, we report that the majority of patients received appropriate antibiotics within 2 h, which is shorter than in many other studies reporting 3 h or longer (19, 22). This, and the fact that 93% of the patients in our cohort were given adequate antibiotics from the onset could partly explain the low mortality rate. So far, Sweden has managed to contain antibiotic resistance (35, 36). It is among the countries with the lowest rates of MRSA (<1%) and *E. coli*-producing ESBL (<5%). First-line antibiotics still work – for example, *S. pneumoniae* is routinely treated with penicillin G or V.

A troubling finding was the noted gender difference in time to seeing a clinician and receiving adequate antibiotics, which could not be explained by severity of disease, clinical presentation, diagnoses, or inflammation (i.e., plasma cytokine levels at inclusions). Previous studies have reported a potential impact of gender on mortality in sepsis, but the results are inconsistent: some found a higher risk in men (37), some in women (38), and some found no difference (39). Here we report, for the first time, that gender influences time to antibiotic treatment. Considering the strong link between time to antibiotics and outcome of severe sepsis and septic shock, this is a clinically important finding. In an attempt to seek the underlying reason for this delay in treatment, the female and male cohorts were compared with respect to diagnosis, etiology, and severity of infection but no differences were identified expect the fact that the females were younger. However it should be noted that this subgroup analyses is based on a small patient cohort and the results need to be verified in larger studies.

In summary, the results demonstrate low mortality rates, both short- and long-term, in this patient cohort despite high APACHE II scores and presence of multiple comorbidities. A multivariate analysis revealed that risk factors for a fatal outcome in sepsis was age, cardiac heart failure, immunosuppression, and SOFA score. Our low mortality rates may be explained by the combination of a well-functioning triage system that directs the patient to the right priority group, the presence of an Infectious Disease specialist both in the ER and the ICU, the lack of resistant isolates and short-time to adequate antibiotics together with early aggressive fluid resuscitation. Although the majority of the patients received adequate antibiotics from the beginning, the data suggested that women with community-acquired severe sepsis and septic shock had a delay in antibiotic treatment as compared to men; a concerning observation that warrants further studies.

## Conflict of Interest Statement

The authors declare that the research was conducted in the absence of any commercial or financial relationships that could be construed as a potential conflict of interest.

## References

[1] Annane D Aegerter P Jars-Guincestre MC Guidet B Current epidemiology of septic shock: the CUB-Rea Network. Am J Respir Crit Care Med (2003) 168:165–72 10.1164/rccm.2201087 12851245

[2] Martin GS Mannino DM Eaton S Moss M The epidemiology of sepsis in the United States from 1979 through 2000. N Engl J Med (2003) 348:1546–54 10.1056/NEJMoa022139 12700374

[3] Dombrovskiy VY Martin AA Sunderram J Paz HL Rapid increase in hospitalization and mortality rates for severe sepsis in the United States: a trend analysis from 1993 to 2003. Crit Care Med (2007) 35:1244–50 10.1097/01.CCM.0000261890.41311.E9 17414736

[4] Esteban A Frutos-Vivar F Ferguson ND Penuelas O Lorente JA Gordo F Sepsis incidence and outcome: contrasting the intensive care unit with the hospital ward. Crit Care Med (2007) 35:1284–9 10.1097/01.CCM.0000260960.94300.DE 17414725

[5] Lever A Mackenzie I Sepsis: definition, epidemiology, and diagnosis. BMJ (2007) 335:879–83 10.1136/bmj.39346.495880.AE 17962288 PMC2043413

[6] Warren BL Eid A Singer P Pillay SS Carl P Novak I Caring for the critically ill patient. High-dose antithrombin III in severe sepsis: a randomized controlled trial. JAMA (2001) 286:1869–78 10.1001/jama.286.15.1869 11597289

[7] Dellinger RP Cardiovascular management of septic shock. Crit Care Med (2003) 31:946–55 10.1097/01.CCM.0000057403.73299.A6 12627010

[8] Laterre PF Levy H Clermont G Ball DE Garg R Nelson DR Hospital mortality and resource use in subgroups of the recombinant human activated protein C worldwide evaluation in severe sepsis (PROWESS) trial. Crit Care Med (2004) 32:2207–18 15640632 10.1097/01.ccm.0000145231.71605.d8

[9] Silva E Pedro Mde A Sogayar AC Mohovic T Silva CL Janiszewski M Brazilian sepsis epidemiological study (BASES study). Crit Care (2004) 8:R251–60 10.1186/cc2892 15312226 PMC522852

[10] Sprung CL Annane D Keh D Moreno R Singer M Freivogel K Hydrocortisone therapy for patients with septic shock. N Engl J Med (2008) 358:111–24 10.1056/NEJMoa071366 18184957

[11] Dellinger RP Carlet JM Masur H Gerlach H Calandra T Cohen J Surviving sepsis campaign guidelines for management of severe sepsis and septic shock. Crit Care Med (2004) 32:858–73 10.1097/01.CCM.0000117317.18092.E4 15090974

[12] Dellinger RP Levy MM Carlet JM Bion J Parker MM Jaeschke R Surviving sepsis campaign: international guidelines for management of severe sepsis and septic shock: 2008. Intensive Care Med (2008) 34:17–60 10.1007/s00134-008-1040-9 18058085 PMC2249616

[13] Dellinger RP Levy MM Rhodes A Annane D Gerlach H Opal SM Surviving sepsis campaign: international guidelines for management of severe sepsis and septic shock, 2012. Intensive Care Med (2013) 39:165–228 10.1007/s00134-012-2769-8 23361625 PMC7095153

[14] Townsend SR Schorr C Levy MM Dellinger RP Reducing mortality in severe sepsis: the surviving sepsis campaign. Clin Chest Med (2008) 29:721–33 10.1016/j.ccm.2008.06.011 18954706

[15] Rivers E Nguyen B Havstad S Ressler J Muzzin A Knoblich B Early goal-directed therapy in the treatment of severe sepsis and septic shock. N Engl J Med (2001) 345:1368–77 10.1056/NEJMoa010307 11794169

[16] Jones AE Brown MD Trzeciak S Shapiro NI Garrett JS Heffner AC The effect of a quantitative resuscitation strategy on mortality in patients with sepsis: a meta-analysis. Crit Care Med (2008) 36:2734–9 10.1097/CCM.0b013e318186f839 18766093 PMC2737059

[17] Gaieski DF Mikkelsen ME Band RA Pines JM Massone R Furia FF Impact of time to antibiotics on survival in patients with severe sepsis or septic shock in whom early goal-directed therapy was initiated in the emergency department. Crit Care Med (2010) 38:1045–53 10.1097/CCM.0b013e3181cc4824 20048677

[18] Kumar A Roberts D Wood KE Light B Parrillo JE Sharma S Duration of hypotension before initiation of effective antimicrobial therapy is the critical determinant of survival in human septic shock. Crit Care Med (2006) 34:1589–96 10.1097/01.CCM.0000217961.75225.E9 16625125

[19] Puskarich MA Trzeciak S Shapiro NI Arnold RC Horton JM Studnek JR Association between timing of antibiotic administration and mortality from septic shock in patients treated with a quantitative resuscitation protocol. Crit Care Med (2011) 39:2066–71 10.1097/CCM.0b013e31821e87ab 21572327 PMC3158284

[20] Levy MM Artigas A Phillips GS Rhodes A Beale R Osborn T Outcomes of the surviving sepsis campaign in intensive care units in the USA and Europe: a prospective cohort study. Lancet Infect Dis (2012) 12:919–24 10.1016/S1473-3099(12)70239-6 23103175

[21] Karlsson S Varpula M Ruokonen E Pettila V Parviainen I Ala-Kokko TI Incidence, treatment, and outcome of severe sepsis in ICU-treated adults in Finland: the Finnsepsis study. Intensive Care Med (2007) 33:435–43 10.1007/s00134-006-0504-z 17225161

[22] Varpula M Karlsson S Parviainen I Ruokonen E Pettila V Community-acquired septic shock: early management and outcome in a nationwide study in Finland. Acta Anaesthesiol Scand (2007) 51:1320–6 10.1111/j.1399-6576.2007.01439.x 17944634

[23] Flaatten H Epidemiology of sepsis in Norway in 1999. Crit Care (2004) 8:R180–4 10.1186/cc2867 15312216 PMC522836

[24] Sunden-Cullberg J Norrby-Teglund A Rouhiainen A Rauvala H Herman G Tracey KJ Persistent elevation of high mobility group box-1 protein (HMGB1) in patients with severe sepsis and septic shock. Crit Care Med (2005) 33:564–73 10.1097/01.CCM.0000155991.88802.4D 15753748

[25] Sunden-Cullberg J Nystrom T Lee ML Mullins GE Tokics L Andersson J Pronounced elevation of resistin correlates with severity of disease in severe sepsis and septic shock. Crit Care Med (2007) 35:1536–42 10.1097/01.CCM.0000266536.14736.03 17452927

[26] Bone RC Balk RA Cerra FB Dellinger RP Fein AM Knaus WA Definitions for sepsis and organ failure and guidelines for the use of innovative therapies in sepsis. The ACCP/SCCM Consensus Conference Committee. American College of Chest Physicians/Society of Critical Care Medicine. Chest (1992) 101:1644–55 10.1378/chest.101.6.1644 1303622

[27] Knaus WA Draper EA Wagner DP Zimmerman JE APACHE II: a severity of disease classification system. Crit Care Med (1985) 13:818–29 10.1097/00003246-198510000-00009 3928249

[28] Vincent JL Moreno R Takala J Willatts S De Mendonca A Bruining H The SOFA (sepsis-related organ failure assessment) score to describe organ dysfunction/failure. On behalf of the Working Group on Sepsis-Related Problems of the European Society of Intensive Care Medicine. Intensive Care Med (1996) 22:707–10 10.1007/BF01709751 8844239

[29] Ranieri VM Thompson BT Barie PS Dhainaut JF Douglas IS Finfer S Drotrecogin alfa (activated) in adults with septic shock. N Engl J Med (2012) 366:2055–64 10.1056/NEJMoa1202290 22616830

[30] Valles J Rello J Ochagavia A Garnacho J Alcala MA Community-acquired bloodstream infection in critically ill adult patients: impact of shock and inappropriate antibiotic therapy on survival. Chest (2003) 123:1615–24 10.1378/chest.123.5.1615 12740282

[31] Guidet B Aegerter P Gauzit R Meshaka P Dreyfuss D Incidence and impact of organ dysfunctions associated with sepsis. Chest (2005) 127:942–51 10.1378/chest.127.3.942 15764780

[32] Volakli E Spies C Michalopoulos A Groeneveld AB Sakr Y Vincent JL Infections of respiratory or abdominal origin in ICU patients: what are the differences? Crit Care (2010) 14:R32 10.1186/cc8909 20230620 PMC2887138

[33] Castellanos-Ortega A Suberviola B Garcia-Astudillo LA Holanda MS Ortiz F Llorca J Impact of the surviving sepsis campaign protocols on hospital length of stay and mortality in septic shock patients: results of a three-year follow-up quasi-experimental study. Crit Care Med (2010) 38:1036–43 10.1097/CCM.0b013e3181d455b6 20154597

[34] Puskarich MA Marchick MR Kline JA Steuerwald MT Jones AE One year mortality of patients treated with an emergency department based early goal directed therapy protocol for severe sepsis and septic shock: a before and after study. Crit Care (2009) 13:R167 10.1186/cc8138 19845956 PMC2784398

[35] Hanberger H Erlandsson M Burman LG Cars O Gill H Lindgren S High antibiotic susceptibility among bacterial pathogens in Swedish ICUs. Report from a nation-wide surveillance program using TA90 as a novel index of susceptibility. Scand J Infect Dis (2004) 36:24–30 10.1080/00365540310017429 15000555

[36] Molstad S Cars O Struwe J Strama – a Swedish working model for containment of antibiotic resistance. Euro Surveill (2008) 13:ii:19041 19021951

[37] Adrie C Azoulay E Francais A Clec’h C Darques L Schwebel C Influence of gender on the outcome of severe sepsis: a reappraisal. Chest (2007) 132:1786–93 10.1378/chest.07-0420 17890473

[38] Pietropaoli AP Glance LG Oakes D Fisher SG Gender differences in mortality in patients with severe sepsis or septic shock. Gend Med (2010) 7:422–37 10.1016/j.genm 21056869 PMC3322379

[39] Esper AM Moss M Lewis CA Nisbet R Mannino DM Martin GS The role of infection and comorbidity: factors that influence disparities in sepsis. Crit Care Med (2006) 34:2576–82 10.1097/01.CCM.0000239114.50519.0E 16915108 PMC3926300

